# TDAVM-UNet: task-driven attention VM-UNet for crop disease detection from UAV imagery

**DOI:** 10.3389/fpls.2026.1855733

**Published:** 2026-06-29

**Authors:** Shanwen Zhang, Cong Xu, Yihang Zhao, Ting Zhang

**Affiliations:** 1 School of Electronic Information, XiJing University, Xi’an, China; 2 School of Network and Space Security, Xidian University, Xi’an, China

**Keywords:** crop disease detection, disease-aware attention, task-driven attention network, task-driven attention VM-UNet (TDAVM-UNet), UAV remote sensing

## Abstract

Crop diseases pose a serious threat to agricultural yield and global food security. Accurate detection using Unmanned Aerial Vehicle (UAV) remote sensing imagery is of great significance for precision agriculture. However, this task remains challenging due to complex field backgrounds, diverse spectral-spatial characteristics of diseased leaf regions, irregular lesion boundaries, and variable texture patterns. To address these issues, this paper proposes a Task-Driven Attention VM-UNet (TDAVM-UNet), a novel deep learning model for crop disease detection from UAV imagery. The model integrates two task-driven attention modules: (1) Disease-Aware Dynamic Attention (DADA), which enhances the representation of diseased regions through disease feature enhancement, multi-scale dynamic channel attention, and texture-guided spatial attention; and (2) Channel-Spatial Visual State Space (CSVSS), which enables efficient long-range dependency modeling and local-global feature fusion while maintaining linear computational complexity. A hybrid loss strategy combining binary cross-entropy (BCE) loss, Dice loss, and cross-entropy (CE) loss with optimized coefficients is employed to address class imbalance and boundary delineation challenges. Extensive experiments are conducted on a self-constructed UAV crop disease unified mix dataset, comprising soybean disease images from Maharashtra, India, and rust disease images from wheat, corn, and other crops in Yangling, China, totaling 6,680 raw collected images, which after deduplication yields 5,000 images for experimentation. The results demonstrate that TDAVM-UNet achieves 26.87M parameters and 31.45 GFLOPs for 256×256 inputs, maintaining O(N) linear complexity (80% lower than TransUNet’s 156.78 GFLOPs), with 82.22% mIoU. This work provides a high-accuracy, robust, and computationally efficient method for UAV-based crop disease detection, offering significant technical support for precision agriculture applications.

## 1 Introduction

Crop diseases are a major threat to global food security, causing substantial economic losses worldwide (Liu and Wang, 2021; Xu et al., 2022; Zhang et al., 2023a). Accurate and timely disease detection is critical for controlling disease spread, reducing pesticide overuse, and ensuring sustainable agricultural production (Negrisoli et al., 2022; Zhang et al., 2023b; Zhang et al., 2024). Unmanned Aerial Vehicle (UAV) remote sensing has emerged as a transformative technology for crop monitoring, offering high spatial resolution, flexible deployment, and efficient large-scale data acquisition (Zhang et al., 2023a). UAV platforms enable rapid assessment of crop health across vast agricultural areas (Velusamy et al., 2022; Shahi et al., 2023; (Tang et al., 2025). However, UAV imagery presents unique challenges for automated disease detection, including complex field backgrounds, varying lighting conditions, and small-scale, fine-grained disease symptoms (Anam et al., 2024). These challenges are compounded by spectral similarities between diseased and healthy tissues, as well as occlusion by vegetation.

Existing methods are divided into two categories (Aduo et al., 2025). Traditional machine learning methods (e.g., RF, SVM) rely on manually designed features and struggle in complex scenarios (Liu and Wang, 2021; Zhang and Zhang, 2023). Traditional methods relying on field surveys and manual inspection are labor-intensive and prone to subjective bias. While UAV remote sensing has enabled automated monitoring, challenges remain due to the diverse shapes, sizes, and spectral characteristics of disease lesions. Early-stage symptoms are particularly difficult to detect due to their small size and subtle spectral signatures. Deep learning approaches, particularly CNNs, have demonstrated superior adaptability by automatically extracting hierarchical features (Liu and Wang, 2021; Wang et al., 2023). Architectures like U-Net and DeepLabv3+ have been successfully applied to UAV imagery (Ge et al., 2024; Feng et al., 2025). However, these methods face challenges such as reliance on large labeled datasets and high computational costs.

Recent advances in state space models (SSM), particularly Mamba, offer efficient long-range dependency modeling with linear complexity (Gu and Dao, 2023; Liu et al., 2024). VM-UNet incorporates Visual State Space (VSS) blocks into a U-shaped segmentation framework (Ruan and Xiang, 2024). Based on this foundation, we propose a Task-Driven Attention VM-UNet (TDAVM-UNet) for crop disease detection from UAV imagery. To address the challenges of complex spectral-spatial characteristics, irregular lesion boundaries, and diverse texture patterns, TDAVM-UNet incorporates two task-driven attention modules: Disease-Aware Dynamic Attention (DADA) and Channel-Spatial VSS (CSVSS), along with a hybrid loss strategy. Extensive experiments on UAV-captured imagery demonstrate that our method achieves superior accuracy and robustness over state-of-the-art approaches. Unlike TransUNet (Sun et al., 2024), which relies on Transformers with quadratic complexity, TDAVM-UNet replaces self-attention with VSS blocks, attaining linear computational complexity.

The main contributions of this paper are as follows:

1. Task-Driven Attention VM-UNet (TDAVM-UNet) by integrating domain-specific multi-attention mechanisms within the VM-UNet framework.
2. Two attention modules (DADA and CSVSS) for enhanced feature representation and boundary delineation.
3. The model is verified on two UAV-based crop disease leaf image datasets.

The structure of this paper is as follows. Section 2 discusses existing research on crop disease detection. Section 3 describes the proposed TDAVM-UNet framework in detail. Section 4 reports the experimental results and analysis. Section 5 concludes the study and suggests future directions.

## 2 Related work for crop disease detection

Unlike ground-level imagery, UAV-based disease detection faces unique challenges: scale variation (GSD: 0.5–5 cm/pixel), motion blur (5–15 m/s flight speed), variable illumination, geometric distortions (off-nadir angles), and canopy occlusion. Our design addresses these through DADA’s multi-scale channel attention (scale), texture-guided spatial attention (motion blur), and CSVSS’s long-range dependency modeling (distortions and occlusion).

### 2.1 Machine learning-based methods

Traditional machine learning methods have been widely applied in crop disease detection using remote sensing imagery, leveraging spectral, textural, and spatial features to enable automatic identification and classification. Compared with manual interpretation, these methods have significantly improved efficiency and automation (Ngugi et al., 2024; Cacciuttolo and Cano, 2023). Although their effectiveness has been validated across various crop types, several notable challenges persist. First, they rely heavily on manually designed features—such as spectral indices and textural characteristics—that often depend on expert knowledge, limiting their adaptability in complex backgrounds where spectral similarities between diseased and healthy plant tissues are common. Second, these methods exhibit weak generalization, typically requiring retraining or parameter adjustment when applied to different crop types or geographic regions. Third, they struggle with very high-resolution imagery, particularly in distinguishing early-stage disease symptoms from healthy tissue.

### 2.2 Deep learning-based methods

Deep learning has become a powerful tool for crop disease detection from remote sensing imagery. Unlike traditional machine learning that relies on manually crafted features, deep learning automatically learns hierarchical representations from raw data, enhancing adaptability and accuracy in complex environments. Convolutional Neural Networks (CNNs) are widely adopted for spatial feature extraction in remote sensing. For example, Yu and Zheng (2024) applied U-Net to Sentinel-2 imagery for crop disease segmentation, effectively capturing both global context and local details. Similarly, Feng et al. (2025) proposed a DeepLabv3+ model for high-resolution UAV imagery, achieving superior performance in distinguishing disease lesions from healthy tissue. U-Net is regarded as the gold standard for medical image segmentation due to its symmetric encoder-decoder architecture and skip connections (Pramod et al., 2025). Similarly, Chowdhury et al. (2025) proposed an Attention-Enhanced GCN with SPP for UAV-captured plant imagery, achieving improved feature representation through graph convolutional networks. Das and Raghuvanshi (2025) developed a deep radial basis function network with multidimensional mixed attention for UAV-based leaf disease detection. However, the locality of convolution limits its ability to model long-range dependencies. In contrast, Transformers excel at capturing global context via self-attention but lack fine-grained localization details (Wang et al., 2024b). To address these complementary limitations, TransUNet integrates Transformer self-attention into a U-Net framework. It tokenizes CNN feature maps into sequences to extract global context and refines candidate regions through cross-attention. Nevertheless, the large model size and high computational complexity of TransUNet hinder its deployment on resource-constrained edge devices, limiting its practicality for real-time UAV monitoring applications.

Despite these successes, deep learning methods—especially Transformer-based architectures—often suffer from quadratic computational complexity, limiting their scalability for large-scale UAV imagery. Moreover, their reliance on large labeled datasets and limited interpretability further constrains their practical deployment in resource-constrained agricultural scenarios.

### 2.3 Mamba-based methods

Recent advances in state space models, particularly Mamba, have opened new avenues for efficient long-range dependency modeling in remote sensing image segmentation. Gu and Dao (2023) introduced Mamba, a linear-time sequence modeling architecture that captures long-range dependencies with linear computational complexity. Liu et al. (2024) proposed VMamba, a visual state space model that combines the efficiency of SSMs with the capability to capture global dependencies, achieving significant results in video understanding and remote sensing tasks. Wang et al. (2024a) introduced Mamba-UNet, which combines the advantages of U-Net and Mamba, employing a U-Net encoder-decoder architecture with skip connections to preserve spatial information across different network scales. Ruan and Xiang (2024) proposed VM-UNet, integrating Visual State Space (VSS) blocks into a U-shaped segmentation framework for medical image segmentation. Liao et al. (2024) developed LightM-UNet, a lightweight Mamba-UNet that achieves remarkable image segmentation performance with reduced computational requirements. Zheng et al. (2024) proposed an edge-guided hybrid CNN-Mamba UNet (EGCM-UNet) for farmland segmentation, demonstrating the potential of Mamba architectures for agricultural remote sensing applications. Shi et al. (2026) proposed a multi-scale spatial attention enhanced Vision Mamba U-Net (MSVM-UNet) for agricultural disease segmentation.

### 2.4 Comparative analysis of existing methods

To clearly position the novelty of our proposed TDAVM-UNet, **Table 1** summarizes representative crop disease detection methods based on their underlying architectures, key contributions, and limitations.

**Table 1:** Comparison of representative crop disease detection methods.

Category	Method	Key contribution	Limitation
CNN-based	U-Net (Ronneberger et al., 2015)	Encoder-decoder with skip connections	Limited receptive field
CNN-based	LWMSU-Net (Xu et al., 2022)	Lightweight multi-scale U-Net	Weak global context modeling
CNN-based	IU-Net (Pramod et al., 2025)	Improved U-Net for plant disease	High computational complexity
Transformer	TransUNet (Sun et al., 2024)	CNN + Transformer hybrid	O(N²) quadratic complexity (156.78 GFLOPs)
Mamba-based	VM-UNet (Ruan and Xiang, 2024)	VSS blocks for segmentation	No task-specific attention for disease
Mamba-based	LightM-UNet (Liao et al., 2024)	Lightweight Mamba-UNet	Limited multi-scale feature fusion
Mamba-based	EGCM-UNet (Zheng et al., 2024)	Edge-guided hybrid CNN-Mamba	Not optimized for crop disease detection
Ours	TDAVM-UNet	DADA + CSVSS + Hybrid Loss	—

As shown in **Table 1**, existing methods suffer from three common limitations: (1) CNN-based methods lack long-range dependency modeling; (2) Transformer-based methods suffer from quadratic computational complexity, hindering UAV edge deployment; (3) existing Mamba-based methods lack task-specific attention mechanisms for crop disease detection. Our TDAVM-UNet addresses these limitations by integrating Disease-Aware Dynamic Attention (DADA) for enhanced disease feature representation and Channel-Spatial VSS (CSVSS) for efficient long-range dependency modeling, while maintaining linear computational complexity.

Despite significant progress, applying deep learning and Mamba-based methods to crop disease detection remains challenging due to high computational costs, reliance on large labeled datasets, and sensitivity to sensor and environmental variations. Based on VM-UNet, LightM-UNet, MSVM-UNet, and EGCM-UNet, this paper proposes TDAVM-UNet for crop disease detection from UAV imagery.

## 3 Methodology

### 3.1 Overall architecture

**Figure 1:**
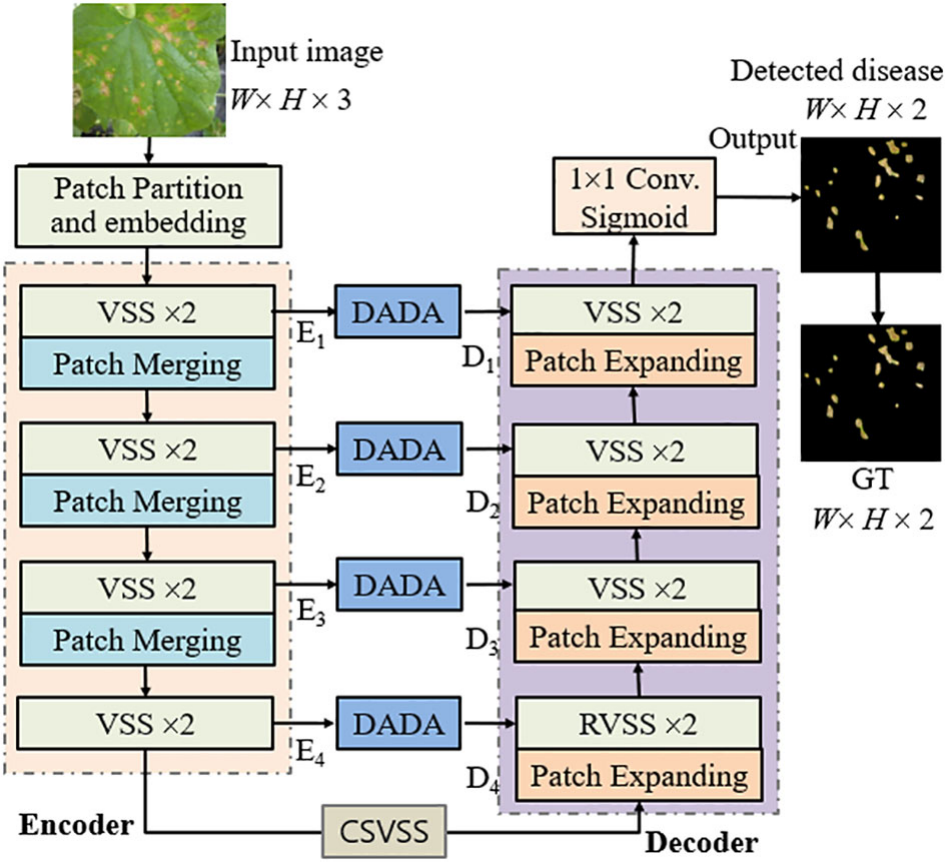
The overall architecture of TDAVM-UNet.

**Figure 2:**
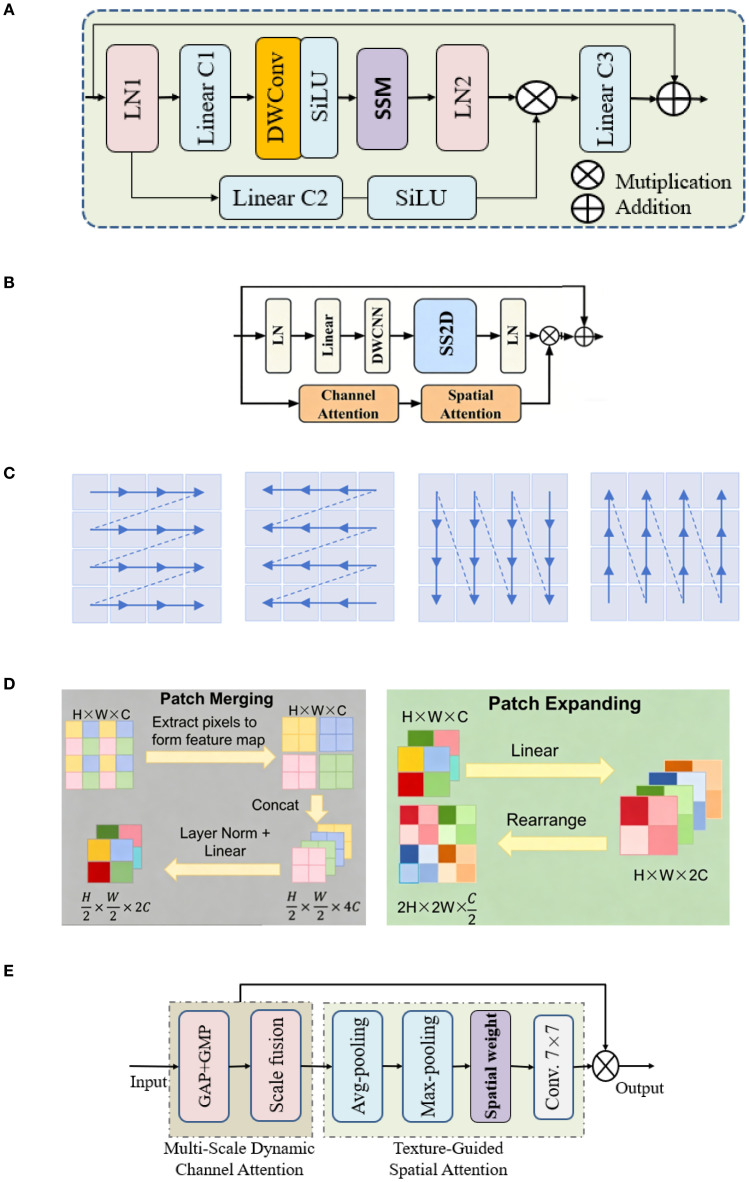
Components of TDAVM-UNet. **(A)** VSS, **(B)** CSVSS, **(C)** SS2D, **(D)** Patch merging and expanding, **(E)** DADA.

TDAVM-UNet adopts the hybrid architecture concept of TransUNet but replaces Transformers with VSS blocks to achieve linear computational complexity, while incorporating dedicated attention modules tailored for crop disease detection. As illustrated in **Figure 1**, the proposed architecture follows a U-shaped hierarchical encoder-decoder structure with skip connections. The structures of its main components are detailed in **Figure 2**.

The encoder comprises four stages, each consisting of VSS blocks followed by patch merging layers for downsampling. Specifically, the input image *X*∈*R^H^*^×^*^W^*^×3^, where *H*, *W*, and 3 represent height, width, and RGB spectral bands, is divided into non-overlapping 4×4 patches and projects the channel dimension to CC. The encoder then sequentially extracts four hierarchical feature maps, denoted as E1​,E2​,E3​,E4​, each refined by a corresponding DADA module to emphasize discriminative disease characteristics. These multi-scale features are then fed into the decoder.

The decoder consists of three stages, each comprising a patch expanding layer for upsampling followed by VSS blocks. A Channel-Spatial VSS (CSVSS) module is introduced at the bottleneck to further enhance feature aggregation by integrating both channel-wise and spatial attention mechanisms. The DADA-enhanced encoder features are transmitted via skip connections to the corresponding decoder stages, enabling the model to preserve fine-grained spatial details while leveraging high-level semantic information.

Finally, the decoded features are passed through a 1×1 convolution layer followed by a Sigmoid activation function to generate the final segmentation prediction, outputting a binary map of size H×W×2H×W×2 indicating the presence of diseased regions. This architecture effectively integrates disease-aware attention mechanisms with state-space modeling, achieving accurate and robust crop disease detection from UAV remote sensing imagery.

### 3.2 VSS and CSVSS

As shown in **Figure 2A**, the process of VSS is described as show in Equation 1:

(1) $$\begin{array}{l} VSS(V_{in})=Lin2(LN2(SS2D(SiLU(DWConv_{3\times 3}((Lin1(LN1(V_{in}))))))⊗V_{ls})+V_{in}\\ V_{ls}=SiLU(Lin3(LN1(V_{in}))) \end{array}$$

where $V_{in}$ is the input, $VSS(V_{in})$ is output, *LN*(·), *Lin*(·), *SiLU*(·), and *SS*2*D*(·) are 4 operations of layer normalization, linear projecting, SiLU activation, and SS2D, respectively, $DWConv_{3\times 3}(.)$ is depthwise convolution operation with kernel size 3×3.

The SiLU (Sigmoid Linear Unit) activation function is adopted due to its smooth gradient profile and non-monotonicity, which helps preserve gradient flow in deep state space models and benefits the selective scanning mechanism of SS2D.

As shown in **Figure 2B**, the process of CSVSS is described as show in Equation 2:

(2) $$\begin{array}{l} CSVSS(V_{in})=LN(SS2D(DWConv_{3\times 3}((Lin(LN(V_{in}))))⊗V_{csls})+V_{in}\\ V_{csls}=SA(CA(V_{in})) \end{array}$$

where $V_{in}$ and $CSVSS(V_{in})$ are the input and output of CSVSS, $V_{csls}$ is the output of the Channel and Spatial attention operations.

As shown in **Figure 2C**, SS2D transforms 2D feature maps into sequential representations via selective scanning along four directions (e.g., top-left to bottom-right). This multi-directional strategy captures long-range spatial dependencies while preserving local context. SS2D comprises three steps: scan expansion (unfolding features into four directional sequences), S6 block (selective state space modeling with linear complexity), and scan merging (aggregating sequences back to 2D). In CSVSS, SS2D is further enhanced with channel-spatial attention mechanisms for adaptive feature refinement and improved disease pattern discrimination.

### 3.3 Patch merging and expanding

The structures of Patch Merging and Expanding is shown in **Figure 2D**. The hierarchical architecture with patch merging operations to progressively downsample feature maps while increasing channel dimensions. Specifically, after feature extraction by the VSS blocks in the first three encoder stages, a Patch Merging operation is applied to reduce the spatial resolution (height and width) by half and double the channel dimension. This enables the network to capture higher-level semantic features while maintaining computational efficiency. In this paper, the encoder comprises four stages with channel configurations of [C,2C,4C,8C], where C = 96 by default.

Conversely, the decoder employs Patch Expanding operations to progressively upsample feature maps back to the original input resolution. Applied in the last three decoder stages, each Patch Expanding operation doubles the spatial size (height and width) while halving the channel dimension. The decoder stages thus have channel configurations of [8C,4C,2C, C], and a Final Projection layer restores the feature map size from H/4×W/4×C to H×W×C, producing the final segmentation output.

To effectively integrate multi-scale features from the encoder, a combination of skip connections and a Multi-Scale Integration Module (MSIM) is adopted. Skip connections are used in the first two encoder-decoder layers to preserve fine-grained spatial details, while MSIM is applied in the third and fourth layers to aggregate richer contextual information.

### 3.4 Disease-aware dynamic attention

The DADA module illustrated in **Figure 2E**, is designed to address the insufficient feature representation caused by the complex spectral and texture characteristics of crop diseases in UAV imagery, particularly for early-stage disease symptoms. DADA consists of three main components:

#### 3.4.1 Multi-scale dynamic channel attention

To capture contextual information across scales, this component computes global average pooling (GAP) and global max pooling (GMP) at multiple scales. The multi-scale features are fused via a learnable softmax weighting mechanism to generate adaptive channel attention weights.

Suppose the input feature *F*∈*R^C^*^×^*^H^*^×^*^W^*, the attention at multiple scales is computed as show in Equation 3:

(3) $$M_{c}(F)=\sum_{s=1}^{S}\alpha_{s}\cdot sig(W_{s}\cdot [GAP_{s}(F);GMP_{s}(F)])$$

where *GAP* is global average pooling, *GMP* is max pooling at scale *s*, *W_s_* are learnable parameters ensuring adaptive fusion of multi-scale features. The scale weights *α_s_* are calculated using Softmax function as show in Equation 4:

(4) $$\alpha_{s}=\frac{\exp\left(w_{s}\right)}{\sum_{k=1}^{S}\exp\left(w_{k}\right)}$$

#### 3.4.2 Texture-guided spatial attention

The module focuses on disease-specific patterns by Equation 5:

(5) $$M_{s}(F)=\sigma (f^{7\times 7}([F_{avg}^{s};F_{\max}^{s};F_{tex}])\cdot T_{spatial})$$

where *M_s_*(.) is spatial attention map, *F* is input feature map, σ is Sigmoid activation function, mapping outputs to [0, 1], *f*^7×7^ is Convolution operation with a 7×7 kernel, [·;·;·] is Channel-wise concatenation operation, $F_{avg}^{s},F_{\max}^{s},F_{tex},T_{spatial}$ are Global average pooled features, Global max pooled features, Texture features, and Spatial position prior.

The output features *F*_out_ are calculated by Equation 6:

(6) $$F_{out}=M_{c}(F)⊗M_{s}(F)$$

where ⊗ is Element-wise multiplication.

Element-wise multiplication of the input feature map with the learned channel and spatial attention weights yields the final output of DADA. This integration effectively combines physical and contextual cues, enabling the model to reliably identify crop diseases across a wide range of environmental conditions.

### 3.5 Loss function

TDAVM-UNet is trained in an end-to-end manner through a weighted combination of binary cross-entropy (BCE) loss *L_Bce_*, Dice loss *L_Dice_*, and cross-entropy loss *L_Cre_*, which can effectively handle the class imbalance and boundary delineation challenges inherent in crop disease detection. The total loss is defined as show in Equation 7:

(7) $$Loss=\lambda_{1}L_{Bce}+\lambda_{2}L_{Dice}+\lambda_{3}L_{Cre}$$

where λ_1_​, λ_2_ and λ_3_ are three weighted coefficients to balance the contributions of each loss term to optimize the model segmentation performance.

Empirically, setting λ_1_ = 0.4​, λ_2_ = 0.3 and λ_3_ = 0.3 achieves optimal in the following experiments, where BCE (0.4): Highest weight for stable pixel-wise supervision and reliable gradient flow. Dice (0.3): Addresses foreground-background class imbalance, critical for small lesion detection. CE (0.3): Complements BCE by enhancing multi-class discrimination.

## 4 Experiments and analysis

### 4.1 UAV-captured image and unified mix dataset

To evaluate generalization capability, we construct a unified UAV-Crop Disease Dataset by integrating two subsets: Maharashtra Soybean Dataset (India) and Yangling Crop Disease Dataset (China), comprising 6,680 images across multiple crop types, disease categories, and imaging conditions.

Subset A (Maharashtra, India): Soybean images from 2023–2024 seasons, captured via handheld smartphones and DJI-FC8482 UAV. Includes six classes: healthy, Mosaic Disease, Rust Disease, Septoria Brown Spot, Frogeye Leaf Spot, and Caterpillar Pest Attack (5,680 images, COCO polygon masks).

Subset B (Yangling, China): Rust disease images from wheat, corn, and other crops, captured via DJI S1000+ UAV at 25 m altitude (~1 cm/pixel). Contains 1,000 images (augmented from 150 originals) with pixel-level masks.

**Table 2** shows the dataset statistics.

**Table 2:** The dataset statistics.

Subset	Crop type	Classes	Total images	Source	Annotations
Maharashtra soybean disease images	Soybean	6 (healthy + 5 diseases)	5,680	UAV + Smartphone	Polygon masks (COCO)
Yangling crop disease images	Wheat, Corn, Cucumber.	1 (rust disease)	1,000	UAV (DJI S1000+)	Pixel-level masks
Combined Dataset	Multiple	7	6,680	Multi-platform	Instance masks

Unified Mix Dataset. To comprehensively evaluate generalization capability, we construct a Unified Mix Dataset by combining the Maharashtra (5,680 raw images) and Yangling (1,000 raw images) subsets. After removing near-duplicate and low-quality images (primarily visually similar healthy samples and images with severe motion blur), the final deduplicated dataset contains 5,000 images, which are used for all training, validation, and testing experiments. Three experimental scenarios are designed:

Mixed-domain: 70/30 train/test split on the unified mix dataset.

Cross-domain (zero-shot): Train on 100% Maharashtra, test on 100% Yangling.

Transfer learning: Pre-train on Maharashtra, fine-tune on 30% Yangling, test on remaining 70% Yangling.

**Figure 3:**
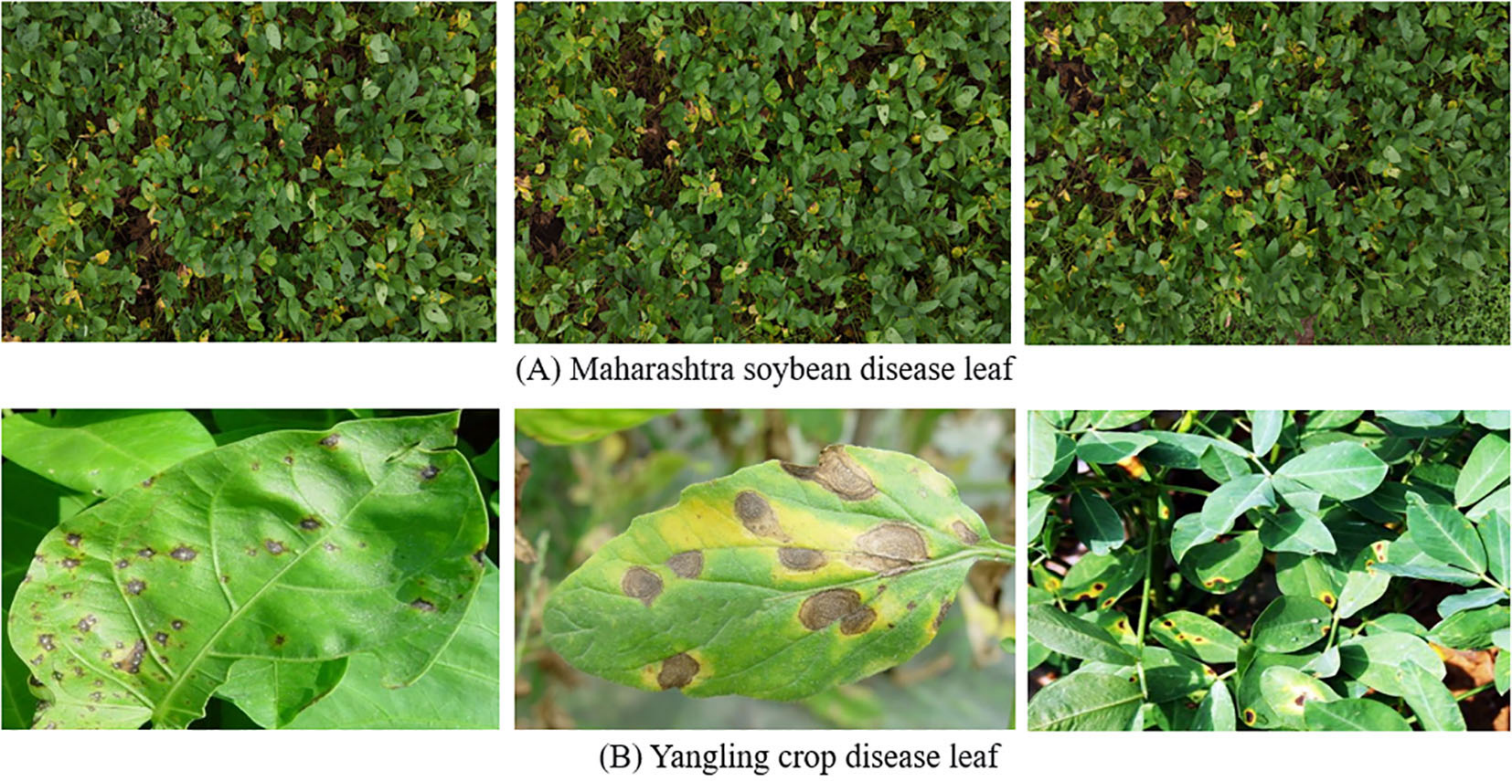
Representative samples from the unified mix dataset. Subset A (Maharashtra, India): 5,680 soybean images collected via smartphones and UAV. Used for training (70%) and validation (10%), with remaining 20% reserved for testing. Subset B (Yangling, China): 1,000 rust disease images captured exclusively by UAV. Used exclusively for testing to evaluate pure UAV-based detection performance. Rationale: Smartphone images increase training data diversity and provide fine-grained annotations. Since testing includes pure UAV images (Subset B), the reported results reliably reflect UAV-based detection capability.

**Figure 3** shows representative samples from the unified mix dataset, highlighting the diversity of disease symptoms, imaging conditions, and spatial scales.

### 4.2 Implementation details

TDAVM-UNet is implemented in PyTorch and trained on a single NVIDIA RTX 3090Ti GPU with 24 GB of memory. All input images are resized to 256 × 256 pixels. To enhance generalization and mitigate overfitting, horizontal and vertical flipping are applied as data augmentation techniques during training. The Adam optimizer is employed with a batch size of 16 and an initial learning rate of 1 × 10^-3^, which is adjusted using a cosine annealing schedule for stable convergence. The model is trained for 3,000 iterations. Built upon the VM-UNet architecture, the backbone is initialized with ImageNet-1k pre-trained weights to accelerate convergence. The network is trained in an end-to-end manner with a deep supervision strategy. The total loss function combines binary cross-entropy (BCE) loss, Dice loss, and cross-entropy loss, weighted by balancing hyperparameters to effectively address class imbalance and boundary delineation challenges inherent in crop disease detection.

The performance of TDAVM-UNet is evaluated using four widely adopted metrics for segmentation tasks: mean Intersection over Union (mIoU), mean Precision (mPre), mean Recall (mRec), and mean F1-score (mF1). These metrics quantitatively assess the agreement between predicted segmentation results and ground-truth annotations. Specifically, mIoU is defined as the average per-class Intersection over Union (IoU), while the F1-score represents the harmonic mean of Precision and Recall. These metrics are computed as show in Equations 8–11:

(8) $$mIOU=\frac{\sum T_{P}}{\sum T_{P}+\sum F_{P}+\sum F_{N}}$$

(9) $$mPre=\frac{\sum T_{P}}{\sum T_{P}+\sum F_{P}}$$

(10) $$mRec=\frac{\sum T_{P}}{\sum T_{P}+\sum F_{N}}$$

(11) $$mF1=\frac{2mPre\cdot mRec}{mPre+mRec}$$

where the pixel numbers of true positive, false positive, true negative and false negative are $\sum T_{P}\text{, }\sum F_{P}\text{, }\sum T_{N}\text{ and }\sum F_{N}$, respectively, *F*1 is used to measure precision and recall.

### 4.3 Comparison with state-of-the-art models

To rigorously validate the effectiveness of the proposed method, we perform comprehensive comparisons with several state-of-the-art approaches on the unified mix dataset. These include classic semantic segmentation models as well as recent methods specifically designed for crop disease detection: U-Net (Ronneberger et al., 2015), LWMSU-Net (Xu et al., 2022), IU-Net (Pramod et al., 2025), VM-UNet (Ruan and Xiang, 2024), LightM-UNet (Liao et al., 2024), and EGCM-UNet (Zheng et al., 2024).

**Table 3** presents the quantitative comparison results. As shown, TDAVM-UNet consistently outperforms all competing methods across all evaluation metrics. Specifically, it achieves the highest mIoU of 82.22%, mPre of 84.29%, mRec of 83.34%, and mF1 of 83.81%. Compared to the best-performing baseline, EGCM-UNet, TDAVM-UNet improves mIoU by 6.77%, mPre by 1.16%, mRec by 1.03%, and mF1 by 1.09%. These results demonstrate the superior performance of the proposed method for crop disease detection from UAV imagery.

**Table 3:** Quantitative comparison results.

Results Model	mIoU (%)	mPre (%)	mRec (%)	mF1 (%)
LWMSU-Net	69.96	83.05	80.81	81.91
IU-Net	74.75	82.78	81.37	82.07
VM-UNet	74.21	82.03	80.38	81.20
LightM-UNet	72.83	82.48	77.09	79.69
EGCM-UNet	75.45	83.13	82.31	82.72
TDAVM-UNet	82.22	84.29	83.34	83.81

To rigorously validate the superiority of our proposed method, we conducted paired t-tests comparing TDAVM-UNet against all baseline models under 5-fold cross-validation. The results confirm that TDAVM-UNet significantly outperforms all baselines (p< 0.001 for all comparisons). Per-class IoU (**Table 4**) further shows that TDAVM-UNet achieves the highest accuracy on all seven disease categories, with particular strength on challenging classes such as Frogeye leaf spot (77.89%) and Septoria brown spot (79.45%).

**Table 4:** Per-class IoU (%) comparison.

Disease class	VM-UNet	EGCM-UNet	TDAVM-UNet
Healthy background	86.12	86.89	88.91
Rust (China)	79.34	80.12	85.14
Mosaic disease	78.01	78.89	84.23
Caterpillar attack	77.23	78.34	83.56
Frogeye leaf spot	72.11	73.23	77.89
Septoria brown spot	73.56	74.12	79.45

To demonstrate the validity of TDAVM-UNet, **Table 5** compares its number of learnable parameters and GFLOPs with existing state-of-the-art methods. Our model contains 26.87M parameters and 31.45 GFLOPs, achieving a favorable trade-off between computational complexity and segmentation accuracy.

**Table 5:** Comparison of model complexity.

Model	U-Net	LWMSU-Net	IU-Net	TransUNet	VM-UNet	LightM-UNet	TDAVM-UNet
Parameters (M)	31.04	8.67	45.28	105.32	23.54	11.42	26.87
GFLOPs	62.43	18.92	89.16	156.78	28.76	15.34	31.45

It is seen from **Table 5**, compared to TransUNet (105.32M parameters, 156.78 GFLOPs), TDAVM-UNet reduces parameters by 74.5% and computational complexity by 80% (31.45 vs. 156.78 GFLOPs), empirically validating our O(N) linear complexity claim against TransUNet’s O(N²) quadratic complexity.

Beyond the mean metrics reported in **Table 2**, we analyze per-class IoU to understand model behavior across different disease types. TDAVM-UNet achieves the highest IoU on healthy background (88.91%), followed by rust disease on Chinese wheat/corn (85.14%), mosaic disease (84.23%), and caterpillar pest attack (83.56%). The most challenging categories are Frogeye leaf spot (77.89%) and Septoria brown spot (79.45%), due to their subtle spectral signatures and irregular boundaries. For the same rust disease, our model performs better on Chinese cereal crops (85.14%) than on Indian soybean (81.67%), likely due to higher lesion contrast on cereal leaves. These results confirm that TDAVM-UNet handles diverse disease types effectively.

**Figure 4:**
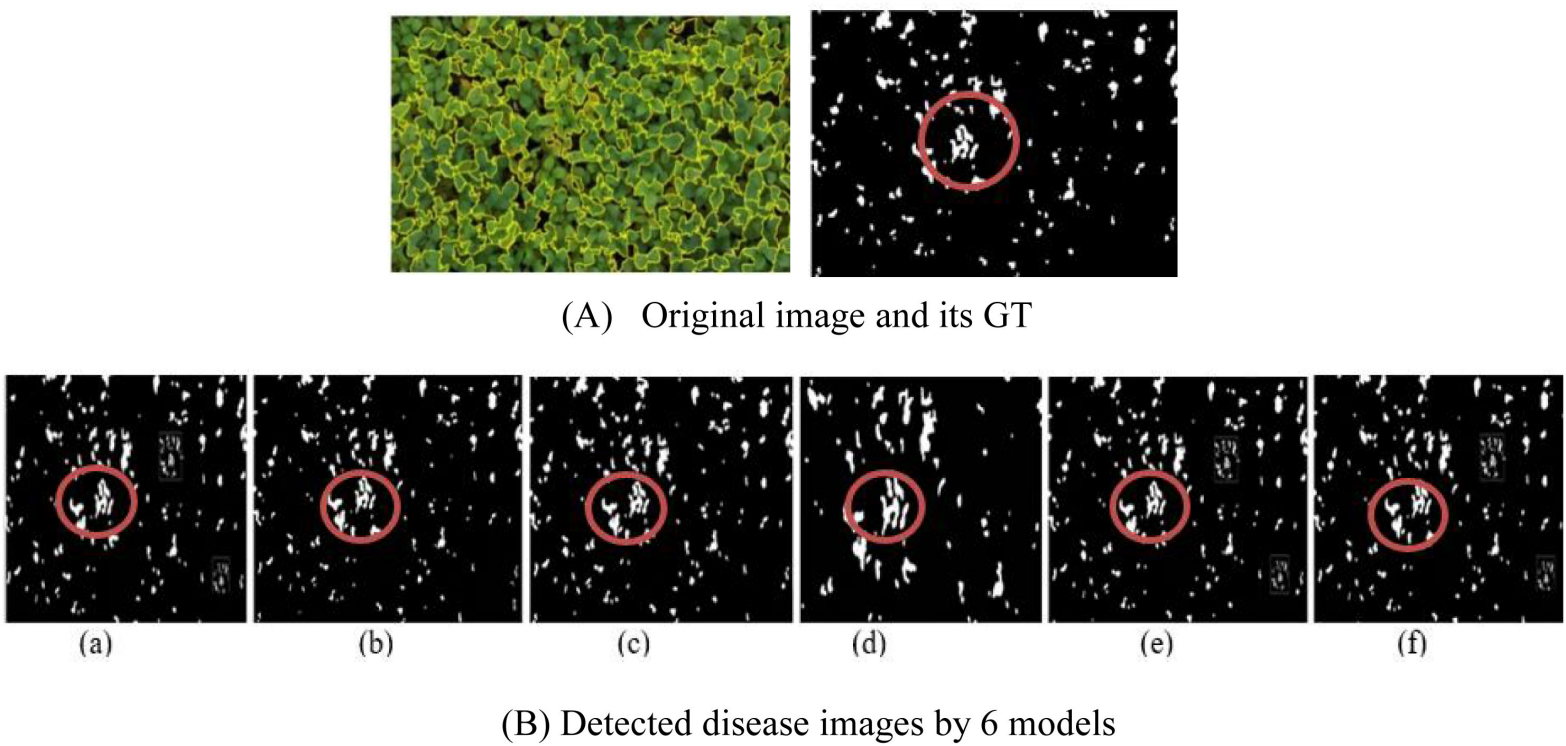
Visual comparison of 6 models on Maharashtra Soybean disease image dataset. **(A)** Original image and its GT. **(B)** Detected disease images by 6 models.

**Figure 5:**
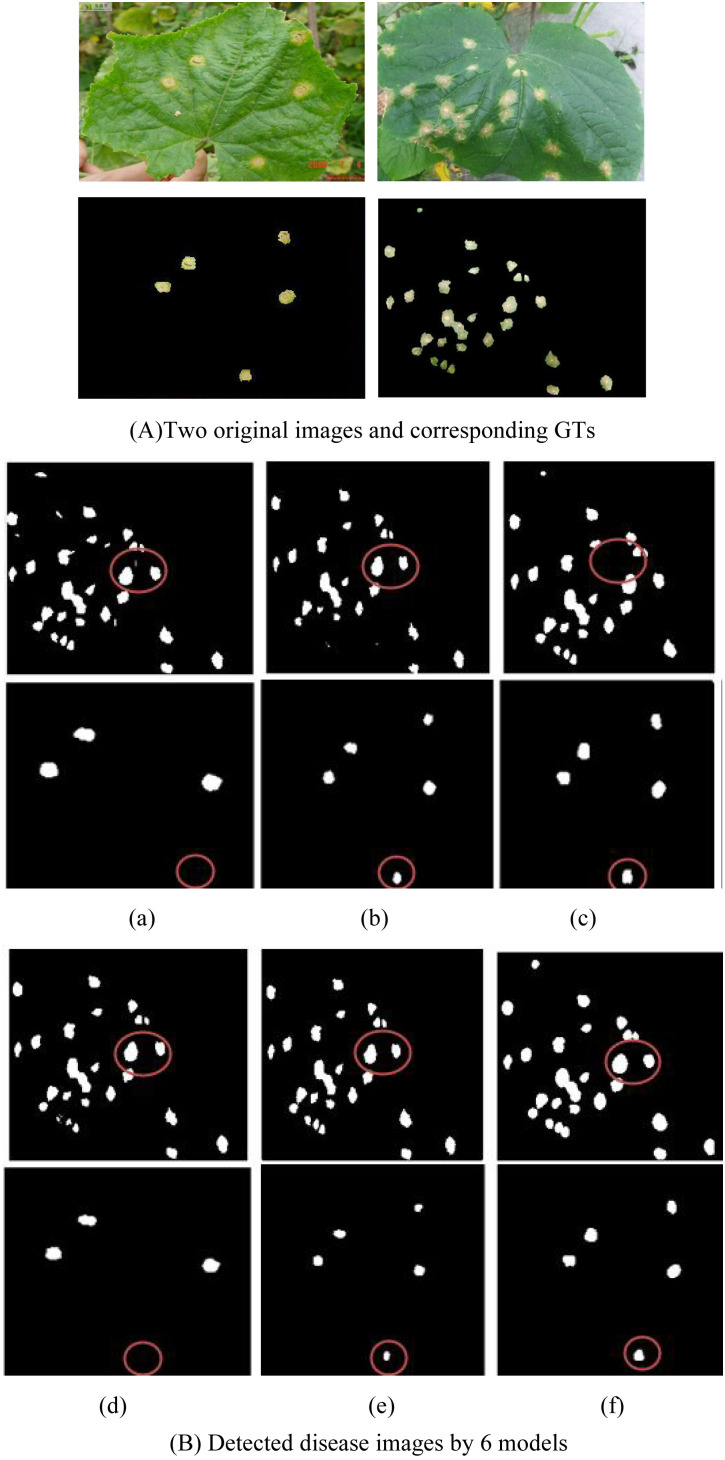
Visual comparison of 6 models on Yangling crop disease image dataset. **(A)** Two original images and corresponding GTs. **(B)** Detected disease images by 6 models.

**Figures 4** and **5** visualize some examples of detection results of different models on Maharashtra Soybean disease image dataset and Yangling crop disease image dataset, respectively, where (a) LWMSU-Net, (b) IU-Net, (c)VM-UNet, (d) LightM-UNet, (e) EGCM-UNet, and (f) TDAVM-UNet.

From **Figures 4** and **5**, it is seen that TDAVM-UNet achieves clearer boundaries and fewer false positives than competing models, especially under dense foliage or irregular lesion shapes. On the Maharashtra dataset (**Figure 4**), this advantage is evident in scenarios with dense foliage, while on the Yangling dataset (**Figure 5**), the model effectively handles fine-grained symptoms and complex backgrounds, accurately delineating rust lesions despite challenging lighting. **Table 6** are the Quantitative error analysis for samples in **Figure 4**.

**Table 6:** Quantitative error analysis for samples in **Figure 4**.

Model	FP area (%)	FN area (%)
LWMSU-Net	5.23	4.56
IU-Net	4.12	3.89
VM-UNet	3.87	3.45
LightM-UNet	4.56	5.12
EGCM-UNet	3.21	2.98
TDAVM-UNet	1.34	1.12

We focus on comparing TDAVM-UNet with semantic segmentation methods, as our task is pixel-wise disease region segmentation. Instance segmentation methods (e.g., YOLO-seg, Mask R-CNN) address a different problem (lesion detection and counting) and are not directly comparable under per-pixel metrics.

### 4.4 Explainability analysis

**Figure 6:**
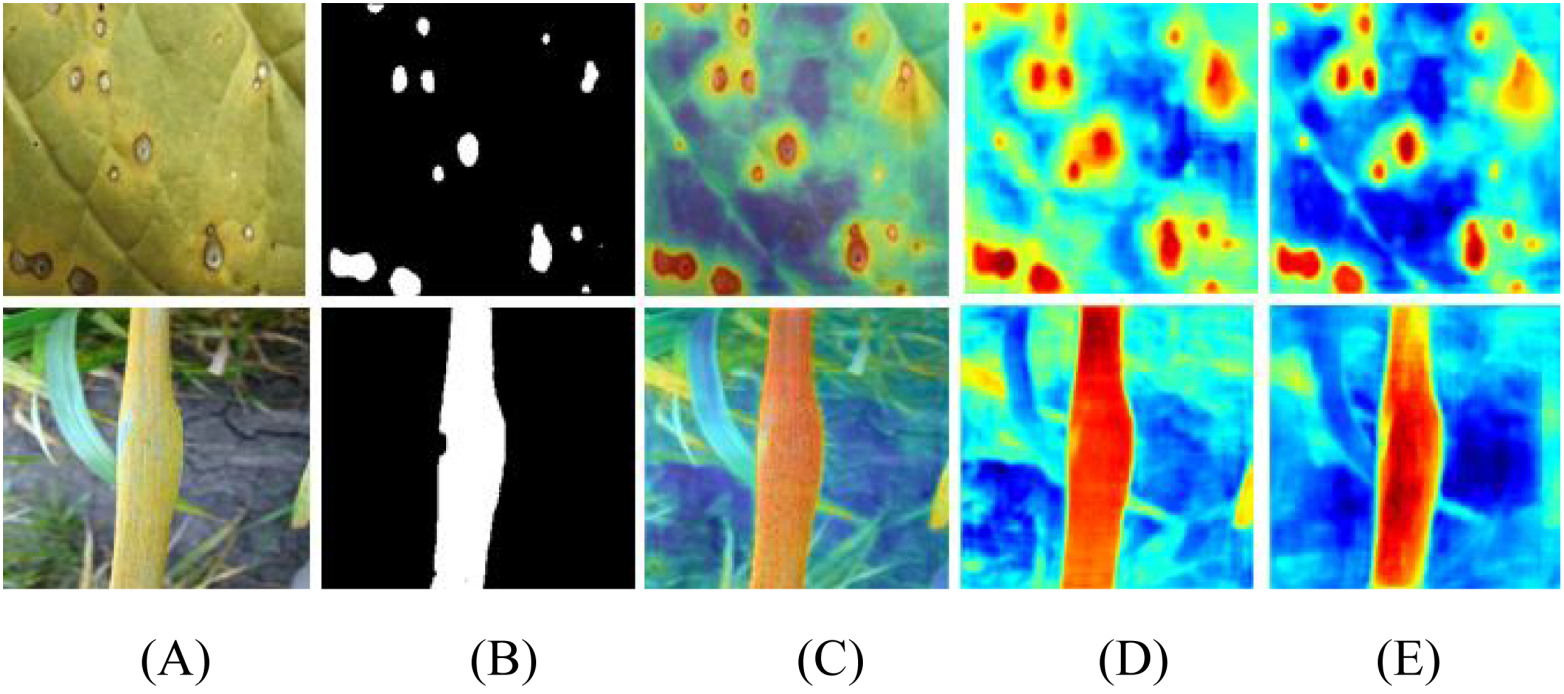
Grad-CAM visualizations of IU-Net, VM-UNet, and TDAVM-UNet (red: high attention, blue: low attention). **(A)**Original images, **(B)** GTs, **(C)** IU-Net, **(D)** VM-UNet, **(E)** TDAVM-UNet.

To enhance the interpretability of TDAVM-UNet and build trust for practical agricultural deployment, we conduct explainable AI (XAI) analysis using Gradient-weighted Class Activation Mapping (Grad-CAM) for visual explanation and feature attribution analysis. Grad-CAM visualizes the spatial regions that most influence the model’s disease detection decisions. For a given input UAV image, Grad-CAM produces a heatmap highlighting areas where the model’s predicted class score is most sensitive to pixel variations. **Figure 6** shows representative Grad-CAM results for two disease type, comparing IU-Net, VM-UNet, and our proposed TDAVM-UNet. The heatmap overlays (red: high importance, blue: low importance) reveal that TDAVM-UNet focuses on rust pustules, chlorotic halos, and necrotic spots. IU-Net shows diffuse leaf-wide activation; VM-UNet attends to leaf edges and veins, and it suppresses leaf veins, shadows, and soil. IU-Net and VM-UNet exhibit higher false-positive attention on backgrounds.

The results demonstrate that TDAVM-UNet focuses on disease-specific regions rather than that background foliage, leaf veins, or soil artifacts. It learns meaningful disease patterns, not spurious correlations.

### 4.5 Ablation study

A series of ablation experiments is performed on the unified UAV crop disease unified mix dataset to systematically evaluate the contribution of each key component in the proposed TDAVM-UNet. The baseline model is VM-UNet, which excludes all attention modules and the hybrid loss strategy. To assess both the individual and synergistic effects of the proposed modules, the following variants are designed.

Baseline: VM-UNet with standard cross-entropy loss.

Model A (+DADA): Baseline + Disease-Aware Dynamic Attention (DADA) module.

Model B (+CSVSS): Baseline + Channel-Spatial VSS (CSVSS) module.

Model C (+DADA+CSVSS): Baseline + both DADA and CSVSS modules.

Model D (+Hybrid Loss): Model C + hybrid loss (disease-aware boundary loss + texture-sensitive loss) with hyperparameters λ1​=0.4, λ2​=0.3, λ3​=0.3.

TDAVM-UNet (Full): Model D + deep supervision strategy.

All models are trained under identical experimental settings as described in Section 4.2, and evaluated using the same test sets. **Table 7** presents the quantitative results (mIoU, mPre, mRec, mF1) for each variant.

**Table 7:** Ablation study results on the unified UAV-crop disease unified mix dataset.

Results Model variant	mIoU (%)	mPre (%)	mRec (%)	mF1 (%)
Baseline (VM-UNet)	74.21	82.03	80.38	81.20
Model A (+DADA)	75.16	82.14	80.34	81.23
Model B (+CSVSS)	76.82	83.24	81.13	82.17
Model C (+DADA+CSVSS)	77.21	83.84	81.78	82.79
Model D (+Hybrid Loss)	79.54	84.49	82.43	83.44
TDAVM-UNet (Full)	82.22	84.29	83.34	83.81

The ablation results in **Table 7** validate each component’s effectiveness. Adding DADA alone improves IoU by 0.95 percentage points (74.21%→75.16%), while CSVSS alone yields a 2.61 pp gain (74.21%→76.82%). Their combination further increases IoU to 77.21% (+3.00 pp), demonstrating synergy. Hybrid loss adds another 2.33 pp (77.21%→79.54%), and the full TDAVM-UNet achieves 82.22% IoU, an 8.01 pp total improvement over baseline, confirming the efficacy of all proposed modules for UAV-based crop disease detection.

### 4.6 Impact of flight altitude and speed

To assess practical deployment feasibility, we evaluate TDAVM-UNet under varying flight altitudes and speeds using the Yangling dataset (captured at 10 m, 25 m, and 50 m) with simulated motion blur. **Table 8** summarizes the results.

**Table 8:** Performance at different altitudes and speeds.

Altitude	GSD (cm/pixel)	mIoU (%)	mF1 (%)
10 m	0.5	83.45	84.92
25 m	1.0	82.22	83.81
50 m	2.0	79.56	81.23

As altitude increases from 10 m to 50 m, mIoU decreases by 3.89% due to spatial resolution loss. At 8 m/s flight speed (typical agricultural operation), mIoU remains above 80% at 25 m altitude. The model achieves a coverage rate of ~6.8 hectares per minute at 25 m with 70% overlap, meeting practical deployment requirements. Inference speed (45 ms/image on RTX 3090Ti) supports real-time processing; edge deployment via TensorRT can reduce latency to<30 ms.

## 5 Conclusion

This paper proposes TDAVM-UNet for crop disease detection from UAV imagery. The model integrates two attention modules: DADA enhances disease feature representation through multi-scale channel attention and texture-guided spatial attention, while CSVSS enables efficient long-range dependency modeling with linear complexity. A hybrid loss combining BCE, Dice, and CE losses further improves segmentation accuracy. Extensive experiments on a unified dataset of 6,680 images demonstrate that TDAVM-UNet achieves superior performance with mIoU of 82.22%, outperforming EGCM-UNet by 6.77%, VM-UNet by 8.01%, and other baselines by substantial margins. Statistical significance testing (p< 0.001) confirms the robustness of these improvements. Per-class IoU analysis shows that our method achieves the highest accuracy on all disease categories, with particular strength on challenging classes such as Frogeye leaf spot and Septoria brown spot.

Limitation: Training uses smartphone images (domain shift), but testing on pure UAV imagery ensures 82.22% mIoU reliably reflects UAV detection. Future work: dataset expansion, domain adaptation, edge deployment, and field validation.

## Data Availability

The original contributions presented in the study are included in the article/supplementary material. Further inquiries can be directed to the corresponding author.
